# The Role of Renal Denervation in the Treatment of Hypertension in Canada: A Case-Based Discussion from the Canadian Hypertension Specialists Society

**DOI:** 10.1093/ajh/hpaf236

**Published:** 2025-12-06

**Authors:** Raj S Padwal, Michael Dorsch, Sheldon Tobe, Jennifer Ringrose, Ernesto L Schiffrin, Ross D Feldman, Bhanu Prasad, Alexander A Leung, Lisa Dubrofsky, Karen C Tran, Mina Madan, Lindsay Machan, Nadia Khan

**Affiliations:** Department of Medicine, University of Alberta, Edmonton, AB, Canada; Department of Medicine, University of Alberta, Edmonton, AB, Canada; Division of Nephrology, Department of Medicine, Sunnybrook Health Sciences Centre, Toronto, ON, Canada; Department of Medicine, Northern Ontario School of Medicine, ON, Thunder Bay, Ontario, Canada; Department of Medicine, University of Alberta, Edmonton, AB, Canada; Hypertension and Vascular Research Unit, Lady Davis Institute for Medical Research, Sir Mortimer B. Davis-Jewish General Hospital, McGill University, Montréal, QC, Canada; Department of Medicine, Sir Mortimer B. Davis-Jewish General Hospital, McGill University, Montréal, QC, Canada; Department of Medicine, University of Western Ontario, London, ON, Canada; Section of Nephrology, Department of Medicine, Regina General Hospital, University of Saskatchewan, Regina, SK, Canada; Division of Endocrinology and Metabolism, Department of Medicine, University of Calgary, Calgary, AB, Canada; Department of Community Health Sciences, University of Calgary, Calgary, AB, Canada; Division of Nephrology, Department of Medicine, Women’s College Hospital, Toronto, ON, Canada; Department of Medicine, University of British Columbia, Vancouver, BC, Canada; Division of Cardiology, Department of Medicine, Sunnybrook Health Sciences Centre, University of Toronto, Toronto, ON, Canada; Department of Radiology, University of British Columbia, Vancouver, BC, Canada; Department of Medicine, University of British Columbia, Vancouver, BC, Canada

**Keywords:** renal denervation, resistant hypertension, blood pressure, case report

## Abstract

Over one-third of Canadians with hypertension do not achieve recommended blood pressure (BP) targets despite availability of effective treatments. Renal sympathetic nerve denervation (RDN) is a recently approved, minimally invasive treatment for hypertension being offered in multiple Canadian centers. How best to implement this procedure in contemporary Canadian clinical practice remains unclear. Herein, we provide a Canadian hypertension specialist viewpoint on use of RDN in Canada. We review the rationale for, and evidence supporting, the use of RDN and discuss, using 2 clinical cases, its potential therapeutic role. We note that RDN has effectively lowered BP in multiple, sham-controlled, randomized clinical trials and has a favorable safety profile. Economic models indicate that it is cost-effective in the Canadian context. Conversely, the BP-lowering effect is relatively modest; no well-established method to pre-identify responders exists; cardiovascular endpoint data supporting use of RDN are lacking; and no clear funding model is currently in place in Canada. Accordingly, we suggest that use of RDN be reserved for willing patients with severely elevated BP despite the use of first-line conventional therapies who have had secondary causes excluded. Examples include patients with resistant hypertension or moderate or severe hypertension and multiple drug intolerance syndrome. In view of its recent approval and known operator-dependency, RDN should be offered solely through programmatic, multidisciplinary collaboration between hypertension specialists and experienced interventionalists using a shared decision-making approach with the patient. Funding deployment should target such programs and sites should carefully monitor their outcomes to confirm comparability to the published literature.

## Introduction

Hypertension is a highly prevalent, modifiable cardiovascular risk factor affecting 23% of the Canadian adult population and approximately 1.3 billion individuals globally.^1^^,^^2^ Despite the availability of effective therapies, 35% of Canadians with hypertension do not achieve recommended blood pressure (BP) targets, reinforcing the need for better implementation of currently available treatments and development of additional options.^1^^,^^3^

While most hypertension management is delivered in primary care, hypertension specialists play an important and complimentary role, providing guidance for more complex cases. The Canadian Hypertension Specialists Society (CHESS), established in 2022, is a non-profit currently comprised of >120 physicians from across Canada with specific expertise in the diagnosis and treatment of BP-related disorders. CHESS was created to provide education and mentorship to current and future hypertension specialists, especially for complex or challenging cases. Secondary hypertension, resistant hypertension, multi-drug intolerance, and autonomic dysfunction are common reasons for referral to CHESS member clinics. CHESS conducts monthly virtual meetings to discuss cases, review key topics, and share best practices. A baseline descriptive survey characterizing specialist care in Canada has been published recently.^4^

Renal sympathetic nerve denervation (RDN) is a novel technology recently approved by the Food and Drug Administration and Health Canada for hypertension treatment.^5–7^ Major professional hypertension societies, including those in the United States and European Union have endorsed its use.^8^^,^^9^ RDN is now being offered at multiple tertiary care Canadian hospitals and a recent publication from Toronto-based specialists summarized well the procedure and supportive evidence, and proposed an implementation pathway that included multidisciplinary, team-based care, appropriate initial assessment, shared decision-making, and post-procedure follow-up.^10^ Although experience with performing the latest generation RDN procedure is growing within several CHESS member-affiliated hypertension speciality clinics, considerable uncertainty remains about how to identify optimal candidates and how best to incorporate the procedure contemporary clinical practice.

Our objective herein is to briefly review the rationale for, and the evidence supporting use of, RDN, before discussing in detail, through a case-based approach, our views regarding the role RDN may play in the care of a patient with uncontrolled hypertension.

## Renal denervation: rationale and evidence

Heightened sympathetic nervous system activity (SNS) is implicated as one major contributor to development and propagation of hypertension, particularly in patients with resistant hypertension, chronic kidney disease, and obesity.^11^^,^^12^ Attenuation of SNS activity, initially performed via effective but poorly tolerated surgical sympathectomy procedures in the early 1900s and, later, through use of sympatholytic pharmacotherapies such as beta or alpha adrenergic blockers or centrally acting sympathomimetics, is a well-established treatment strategy to lower BP.^13^^,^^14^

RDN was developed to be a minimally invasive, focused, and tolerable option to reduce SNS activity, with the initial proof-of-principle study reported in 2009.^15^ Conceptually, it is performed by ablating the afferent and efferent sympathetic nerves that lie in the peri-adventitial arterial space exterior to the renal arterial wall while minimizing effects on the arterial wall or within the lumen.^16^ While initial results from open-label trials using office BP as the primary endpoint were promising, the first randomized sham-controlled trial (SYMPLICITY HTN-3) demonstrated overall null results (i.e., no statistically significant sham-subtracted BP reduction), prompting reassessment of the field, and systems redesign.^17^ Potential explanations for the lack of efficacy in SYMPLICITY HTN-3 include incomplete ablation (catheter design, operator inexperience, failure to ablate accessory branches, failure to reach distal arterial segments where denser innervation is present); use of more robust endpoints (24-h ambulatory BP monitoring or ABPM); and enhanced medication effect, with increased medication adherence during trial periods, masking a potentially beneficial effect of RDN.^18^ Additional clinical trials were performed, with several of these newer trials utilizing double-blind, sham-controlled methodologies and including subjects that were not receiving antihypertensive therapy (Table 1).

**Table 1.** Sham-controlled randomized trials of newest generation Food and Drug Administration approved renal denervation systems.

Trial name	Year	Population	Primary endpoint	BP difference (RDN vs Sham)
**Spyral system**				
**SPYRAL HTN OFF-MED Pilot**	2017	80 subjects with untreated, uncontrolled HTN	Change in 24-h systolic BP at 3 months	−5.5 mmHg vs −0.5 mmHg *P* = .04
**SPYRAL HTN OFF-MED Pivotal**	2020	331 subjects with untreated, uncontrolled HTN	Change in 24-h systolic BP at 3 months	−4.7 mmHg vs −0.6 mmHg *P* < .01
**SPYRAL HTN ON-MED Pilot**	2018	80 subjects with uncontrolled HTN on 1-3 medications	Change in 24-h systolic BP at 6 months	−9.0 mmHg vs −1.6 mmHg *P* < .01
**SPYRAL HTN ON-MED Expansion**	2023	257 subjects with uncontrolled HTN on 1-3 medications	Change in 24-h systolic BP at 6 months	−5.9 mmHg vs −5.8 mmHg *P* = .97
**Paradise System**				
**RADIANCE HTN SOLO**	2018	146 subjects with untreated, uncontrolled HTN	Change in daytime systolic BP at 2 months	−8.5 mmHg vs −2.2 mmHg; *P* < .01
**RADIANCE II**	2023	150 subjects with uncontrolled HTN	Change in daytime systolic BP at 2 months	−7.9 mmHg vs −1.8 mmHg *P* < .001
**RADIANCE HTN TRIO**	2021	136 subjects with resistant hypertension on a single pill combination of 3 agents	Change in daytime systolic BP at 2 months	−8.0 mmHg vs −3.0 mmHg *P* = .02

Two methods of performing catheter-based RDN are currently FDA approved.^8^^,^^16^

**Figure 1.**
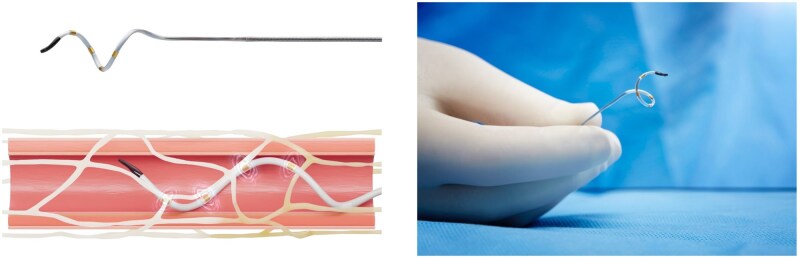
Symplicity spyral renal denervation system. Figure courtesy of Medtronic Canada.

**Figure 2.**
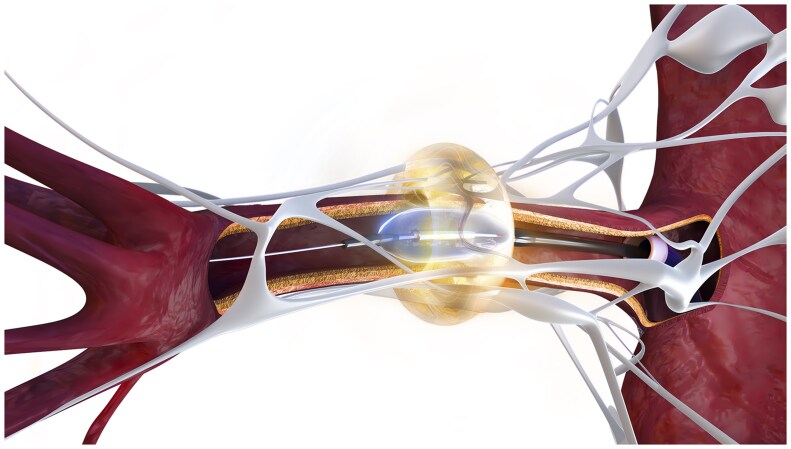
Paradise ultrasound renal denervation system. Figure courtesy of Recor Medical.

1. Radiofrequency-based (Symplicity Spyral System; Medtronic): A quadripolar catheter, with electrodes configured in a helical design (Figure 1), is used to deliver multifocal radiofrequency energy to the peri-adventitial space. Compared to earlier iterations, the current system provides more complete ablation of the renal nerves and major accessory branches.^19^
2. Ultrasound-based (Paradise Ultrasound System; Recor Medical): a cylindrical ultrasound transducer is used to deliver energy in a circumferential, ring-like configuration exterior to the arterial wall (Figure 2). A balloon filled with cooling solution centers the transducer and reduces the temperature within the arterial lumen, preventing injury.

In a meta-analysis of 10 trials comprising 2478 patients with hypertension, it was reported that, compared to sham, RDN reduced 24-h ambulatory systolic BP by 4.4 mmHg (95% CI, −2.7 to −6.1 mmHg), 24-h diastolic BP by 2.6 mmHg (95% CI, −3.6 to −1.5 mmHg), office systolic BP by 6.6 mmHg (95% CI, −3.6 to −9.7 mmHg), and office diastolic BP by 3.5 mmHg (95% CI, −1.6 to −5.4 mmHg) over 2-6 months follow-up.^20^ One issue clouding the interpretation of some trials, and potentially rendering the above summary estimates conservative, is the substantial BP drop observed in the sham arm of some trials. For example, in SYMPLICITY HTN-3, changes in office systolic BP were −14.1 ± 23.9 mmHg in the RDN arm versus −11.7 ± 25.9 mmHg in the sham arm. Thus, the overall BP reduction following RDN was relatively large and of a magnitude expected to reduce long-term cardiovascular events,^21^ but the sham-subtracted difference was small, equal to only −2 mmHg (95% CI, −6.9 to 2.1 mmHg).^17^ Such a large sham response, likely partly attributable to supportive care delivered within the trial and improved adherence, would not be expected to be present in routine clinical care settings. Of note, longer-term observational follow-up studies support durable long-term reductions in systolic BP of 12.7 mmHg (*P* = .05; 36 months; extension of 2 randomized sham-controlled trials) and 14.8 mmHg (*P* = .005; 4 observational studies; mean follow-up 7.7 years).^22–24^

Complications including renal artery stenosis, hypertensive crises, change in renal function, and all-cause death were very infrequent and not different between groups. Change in estimated glomerular filtration rate from baseline was −0.75 mL/min per 1.73 m^2^ (95% CI, −2.0 to 0.5) for RDN and −0.62 mL/min per 1.73 m^2^ (95% CI, −2.2 to 1.0) for sham treatment. In a separate meta-analysis of 50 studies (n = 5769; mean follow-up 6 months), a 0.45% post-RDN renal artery stenosis or dissection incidence was observed, with 24 patients undergoing stenting.^25^

Twenty-four-hour ABPM studies indicate that BP reductions persist throughout the 24-h period (“always-on effect”).^26^ This continuous effect may differ from the more variable BP control that can be seen with pharmacotherapy, and may be beneficial for patients that do not achieve full 24-h control with drug therapy, such as those who are non-adherent, are on short-acting agents, or have nocturnal or early morning hypertension.^26^^,^^27^ Clinical characteristics indicative of heightened SNS activity (i.e., higher resting heart rate, female with abdominal obesity, orthostatic hypertension ≥20/10 mmHg], a higher baseline diastolic BP [>136 mmHg], and greater nighttime BP variability [>12 mmHg]) have each predicted better response to RDN in single studies, but not in a reproducible fashion.^28^ Therefore, no definitive predictor of response has been identified beyond higher baseline systolic BP.^8^^,^^29^^,^^30^

No published trials evaluating the effect of RDN on cardiovascular outcomes exist. In a modeling exercise using data from 2651 patients enrolled in the Global SYMPLICITY Registry (GSR; observational data from 196 centers in 45 countries), based on the modeled benefits from a 14.8-mmHg observed long-term reduction in office BP, it was estimated that RDN would result in 31 major cardiovascular events avoided per 1000 patients treated over a 3-year time frame.^31^ Estimated benefits were greater in patients at higher cardiovascular risk (i.e., those with resistant hypertension, diabetes, chronic kidney disease, or high estimated baseline cardiovascular risk). In addition to the GSR, we note that an analogous registry, the Global Paradise System Registry, has recently been created to collect long-term outcomes for ultrasound-based RDN.^32^

In a recent cost-effectiveness analysis within the Canadian healthcare setting, it was estimated using a Markov model that RDN would add 0.51 (15.81 vs 15.30) quality-adjusted life years compared to standard care, at an incremental cost of $6,031 (RDN: $73,971 vs standard care: $67,040) over a lifetime.^33^ This resulted in an incremental cost-effectiveness ratio of $11,809 per quality-adjusted life year gained, which is considered acceptable relative to commonly cited consensus-based cost-effectiveness thresholds.^33^

## Case one—initial assessment

*A 43-year-old female with a 10-year history of uncontrolled hypertension was referred for assessment in the context of several recent visits to the Emergency Department for severely elevated BP in the 180/100s mmHg range. She reported a history of chronic, intermittent headaches, occasionally associated with elevated heart rate but no other paroxysmal symptoms. Family history was positive for hypertension. Past medical history included a left vertebral artery dissection thought to be related to uncontrolled hypertension and causing a lateral medullary stroke 9 years prior, diabetes, chronic anxiety and depression, chronic right rotator cuff pain, and obstructive sleep apnea on continuous positive airway pressure. BP medications included valsartan-hydrochlorothiazide 320-25 mg daily and nifedipine XL 60 mg daily.*

*Central adiposity, purplish abdominal striae, and a dorsal fat pad but no other Cushingoid features were present on physical examination. BMI was 39 kg/m^2^. EKG was normal. Echocardiogram showed an ejection fraction of 55 and normal left ventricular wall thickness. A1c was 6.4% and creatinine was 69 µmol/L (eGFR 93 mL/min/1.73 m^2^). Albumin-to-creatinine ratio was 3.0 mg/mmol (normal <3.0 mg/mmol). No prior episodes of hypokalemia or metabolic alkalosis were documented.*

*Twenty-four-hour ABPM showed a mean overall BP of 161/104 mmHg, mean awake BP of 165/106 mmHg, and mean nighttime BP of 154/101 mmHg. Mean heart rate was 92 bpm. Non-dipping status, at 7% for systolic and 5% for diastolic, was seen. Mean home BP was 182/119 mmHg.*

*Cardiovascular risk was determined to be high, given the hypertension and non-dipping status, prior stroke, and diabetes (albeit well controlled). She was considered to have resistant hypertension, given the Stage 3 BP elevation despite treatment with 3 optimally dosed medications. Adherence and health behavior modification counseling were given. Proper home BP monitoring technique was reinforced. Screening tests for primary aldosteronism, Cushing’s syndrome (given the physical exam findings), and pheochromocytoma (given her presenting symptoms) were requested. As the pre-test probability for pheochromocytoma was low, bisoprolol was added at a dose of 5 mg daily (given high-normal heart rate), with a plan to add spironolactone if needed once screening for primary aldosteronism had been performed.*

## Resistant hypertension: general approach

**Figure 3.**
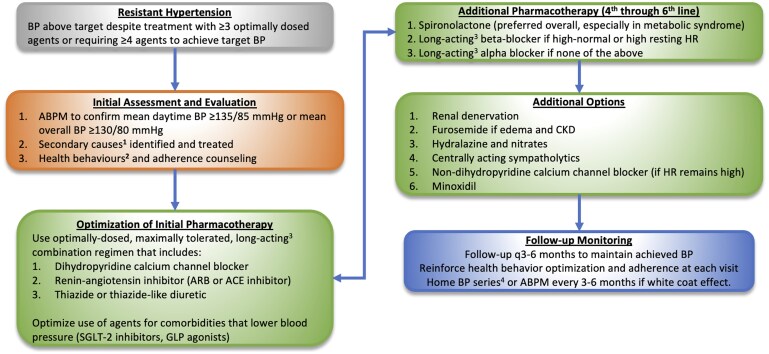
Algorithm for the assessment and management of resistant hypertension. ^1^Screen **all** patients for primary aldosteronism (plasma aldosterone/renin ratio), drug and alcohol use disorder, renal parenchymal disease (urinalysis, serum creatinine, ultrasound) and **selected** patients (based on symptoms and signs) for obstructive sleep apnea (Epworth Scale, overnight polysomnography), renal artery stenosis (CT or MR angiography), pheochromocytoma (plasma or 24-h urinary metanephrines), Cushing’s syndrome (24-h urinary free cortisol, late-night salivary cortisol and/or 1 mg dexamethasone suppression test), hyper/hypothyroidism (TSH), aortic coarctation (thoracic CT or MRI), primary hyperparathyroidism (serum calcium). ^2^Sodium restriction to 2 g per day or less, weight loss to target BMI <25 kg/m^2^, DASH diet, 30-60 min exercise on most days of week, limit excessive alcohol intake, smoking cessation, sleep optimization, stress management. ^3^Long-acting agents include: CCB (amlodipine, long-acting nifedipine), ACE inhibitor (perindopril, trandolapril), ARB (telmisartan, olmesartan, irbesartan), beta-blocker (bisoprolol, long-acting metoprolol), alpha blocker (doxazosin). ^4^Mean of duplicate readings performed in morning and evening for 7 days.

Resistant hypertension, potentially affecting 5% of patients with hypertension,^34–36^ is considered present if BP levels remain uncontrolled despite treatment with 3 optimally dosed antihypertensive drugs, or if 4 or more agents are required to control BP.^36^ Patients often have hypertension that is multifactorial in nature (i.e., primary or polygenic plus central adiposity induced plus/minus another secondary cause). Evaluation of apparent treatment-resistant hypertension should follow a structured approach (Figure 3). Ideally, 24-h ABPM should be performed to rule out white coat effect; if unavailable, the mean BP of a properly collected 7-day home BP series can serve as an alternative. As non-adherence to antihypertensive medications is highly prevalent, patients should be questioned about medication adherence and/or prescription refill data should be reviewed.^37^ Therapeutic drug monitoring is a more direct method of assessing adherence but is largely not available in Canada.^38^ Health behaviors should be optimized (reduce excessive sodium intake, normalize body weight and waist circumference, engage in regular physical activity, smoking cessation, sleep optimization, stress management) and exogenous drugs or medications contributing to elevated BP should be, if possible, minimized or discontinued.^36^ A multidisciplinary approach can be helpful, leveraging the complimentary expertise of nurses (proper BP measurement technique, general patient education), nutritionists (implementing the Dietary Approaches to Stop Hypertension [DASH] diet, reducing sodium intake), pharmacists (adherence assessment and counseling, adverse drug reactions), and physical therapists (exercise counseling and proper implementation).

Work-up for secondary causes in adults should be performed (Figure 3). Key considerations include:

1. Obesity and sleep apnea are common causes of secondary hypertension and weight loss is often effective for reducing BP.
2. All patients should be screened for primary aldosteronism (aldosterone-renin ratio) and renal parenchymal disease (urinalysis, serum creatinine and eGFR, plus/minus abdominal ultrasound).
3. Based on clinical suspicion and symptoms, selected patients should be screened for Cushing’s syndrome (low-dose dexamethasone suppression test, 24-h urinary free cortisol, or late-night salivary cortisol), pheochromocytoma (plasma or urine metanephrines), aortic coarctation (CT angiogram, or magnetic resonance angiography), renal artery stenosis (CT angiogram or magnetic resonance angiography), thyroid disease (TSH), and primary hyperparathyroidism (serum calcium level).^36^

The medication regimen should be adjusted to ensure use of optimally dosed, maximally tolerated, long-acting drugs. The ideal initial regimen consists of a dihydropyridine calcium channel blocker, angiotensin-receptor blocker or angiotensin converting enzyme inhibitor, and thiazide-like diuretic.^39^ Use of combination agents and medications for comorbidities that may also lower BP (e.g., sodium-glucose cotransporter [SGLT2] inhibitors and glucagon-like peptide-1 [GLP] or dual GLP/glucose-dependent insulinotropic peptide [GIP] receptor agonists) should be considered.^36^

If BP remains above target, additional options include spironolactone (preferred, especially if metabolic syndrome is present), beta-blockers (if heart rate is high), centrally acting sympathomimetics or peripheral alpha blockers.^40^ Additional agents that can be considered, as needed (Figure 3) but are generally less well tolerated.

## Case one—management and follow-up

*Primary aldosteronism screening showed the following: aldosterone 153 pmol/L; renin 9.3 ng/L, ratio 16 pmol/ng. Although all 3 antihypertensives can cause false negative results, further testing following rotation to non-interfering medications was not pursued given that the ratio was well under the local threshold of >100-140 pmol/L/ng/L for a positive test. Review of a recent CT scan revealed normal adrenal gland morphology. One milligram dexamethasone suppression test was negative, with a morning cortisol result of 34 nmol/L (normal <50 nmol/L). Plasma metanephrine and normetanephrine levels were 0.13 and 0.36 nmol/L (normal <0.50 and 0.71 nmol/L, respectively).*

*Over several months, bisoprolol was increased to 10 mg, spironolactone was added and titrated to 50 mg daily, and clonidine 0.1 mg twice daily was initiated. Initiating semaglutide led to weight loss of nearly 20 lbs, but was ultimately discontinued due to gastrointestinal side effects. Weight was not regained. Mean home BP dropped to 178/100 mmHg. Pharmacy record review indicated that prescriptions were being filled and it was felt that the patient was adherent.*

*The patient was referred for RDN. CT angiogram of the renal arteries showed normal anatomy and no renal artery stenosis. Fentanyl and midazolam were administered as preprocedural sedation. Ultrasound and fluoroscopic guidance was used to position a 6-French sheath in the right femoral artery. An iliac angiogram confirmed adequate puncture and sheath position. A 55-cm renal double curve catheter was inserted over a standard wire. Pre-ablation renal angiogram confirmed suitability. A 0.014-inch coronary guidewire was placed in the distal right renal artery allowing delivery of the Symplicity Spyral multielectrode RDN radiofrequency ablation catheter into the main renal artery. Multiple successful extra-parenchymal ablations were performed, moving distally to proximally, followed by selective renal angiogram, which confirmed no arterial injury. The process was then successfully repeated on the left side. The arteriotomy site was then closed with an Angio-Seal device.*

*The patient was admitted overnight and previous antihypertensive medications were continued. BP dropped acutely that evening, to a nadir of 100/53 mmHg before rising to 120/73 mmHg. She was discharged the following day, instructed to monitor BP daily, and assessed in clinic 1 week later. She remained off medication because the BP remained relatively low. Mean home BP was 140/89 mmHg and mean heart rate was 90 beats per minute (bpm). Anticipating further BP rise, valsartan hydrochlorothiazide 320-25 mg daily, bisoprolol 5 mg daily, and spironolactone 50 mg daily were restarted. Clonidine and nifedipine were not. She experienced post-procedural bilateral costovertebral angle pain, which gradually resolved over a 3-week span. Over the next several months, BP gradually dropped and spironolactone was discontinued. Seven months post-RDN, she reported a mean home BP level of 119/70 mmHg and mean heart rate of 65 bpm on a regimen of valsartan hydrochlorothiazide 320-25 mg daily and bisoprolol 5 mg daily.*

This case of a patient with multifactorial resistant hypertension (primary, central adiposity, OSA, chronic pain, anxiety) demonstrated good response to RDN, with reduction in drug burden from 5.5 to 2.5 agents and improved BP control. Unanticipated effects included protracted, self-limited back pain and an initial hypotensive response that lasted several days. There was no clear explanation for the latter, although we hypothesize that when antihypertensive drugs are administered in supervised hospital environment, acute and significant drops in BP may occur, especially if patients were previously non-adherent. In addition, perhaps BP may drop because of periprocedural sedation and/or more effective pain management if underlying chronic pain is present. This illustrates the need for frequent, regular follow-up and communication between interventionalists and hypertension specialists to optimize post-procedural BP control and avoid significant BP lability.

## Case two—initial assessment

*A 74-year-old female with a 14-year history of hypertension was referred with inability to tolerate multiple medications and multiple Emergency Department visits for uncontrolled BP. Prior adverse effects were to renin-angiotensin inhibitors (perindopril [tinnitus] and irbesartan [facial swelling]), beta-blockers (propranolol [facial swelling] and carvedilol [sedation]), calcium channel blockers (nifedipine [myalgias and facial flushing] and diltiazem [nightmares]), and thiazides (hydrochlorothiazide [dehydration, with documented hyponatremia]). At the time of initial referral, she was taking telmisartan 40 mg daily, although she reported dry eyes, myalgias, fatigue, and intermittent palpitations from this medication and wished to discontinue it.*

*Past medical history included anxiety disorder and spinal stenosis. She had undergone myocardial perfusion testing 3 years earlier, with a possible small region of apical ischemia being present but no treatment was initiated given medication intolerance. She also reported chronic constitutional symptoms, but rheumatological work-up was negative. Family history was positive for hypertension.*

*Physical examination was unremarkable. BMI was 26 kg/m^2^. EKG showed a left bundle branch block. Ultrasound showed normal sized kidneys. MRI previously showed normal adrenal morphology. Echocardiogram showed an ejection fraction of 51%, a sigmoid-shaped septum, and subtle apical hypokinesis. A1c was 5.6% and creatinine was 74 µmol/L (eGFR rate 69 mL/min/1.73 m^2^). Albumin-to-creatinine ratio was 0.28 mg/mmol (normal <3.0 mg/mmol). CT head showed mild microangiopathic white matter changes consistent with small vessel disease.*

*Twenty-four-hour ABPM was recommended but the patient declined. Mean home BP was 165/85 mmHg. Proper home BP monitoring technique was reinforced and use of a clinically validated device confirmed. Adherence and health behavior modification counseling were given. Screening for primary aldosteronism, performed off antihypertensive medications, was negative (aldosterone 617 pmol/L; renin 8.1 ng/L, ratio 76 pmol/ng). Plasma metanephrine and normetanephrine levels were 0.17 and 0.56 nmol/L (normal <0.50 and 0.71 nmol/L, respectively).*

## Multiple drug intolerance syndrome: general approach

Multiple Drug Intolerance Syndrome (MDIS) is a unique clinical entity characterized by adverse drug reactions to at least 3 unrelated drugs used commonly in a therapeutic area, occurring on different occasions, and not related to an allergic reaction.^41^ It has been described for antibiotics, non-steroidal anti-inflammatory medications, and antihypertensives with an overall prevalence of 2%-10%.^41^ In one cohort of 1000 patients, the prevalence of intolerance to 3 or more antihypertensive drugs was 8%.^42^ Further, BP control rates in patients with MDIS have been reported to be as low as 20%.^43^ Anxiety disorders, somatization, or gastrointestinal comorbidities may be present.^44^

A structured approach to MDIS, characterized by initial fractional dosing with gradual up-titration, use of ultra-low dose drug combinations, and use of liquid and patch-based formulations to avoid reaction to pill-based excipients may improve control.^36^ Psychological counseling or psychiatric evaluation may be of theoretical benefit if uncontrolled anxiety is a major contributor.

## Case two—management and follow-up

*Telmisartan was discontinued. Over 2 years, multiple medications were initiated, typically beginning at quarter-strength dosed every other day. The patient was unable to tolerate each, typically discontinuing within a week. Mean home BP levels varied from 160/90 to 181/100 mmHg, lower on medication. Adverse effects included olmesartan (chest pain), candesartan (headache), spironolactone (confusion), chlorthalidone (documented hyponatremia), clonidine (chest pain), doxazosin (peripheral edema), hydralazine (diarrhea), and nitrates (throat tightness).*

*Given the inability to tolerate pharmacotherapy and high-risk status, the patient was referred for RDN. A pre-procedure mean home BP series was 165/85 mmHg. CT angiogram of the renal arteries showed normal anatomy, with no evidence of renal artery stenosis. The procedure was successfully conducted with no complications. The patient was admitted for overnight observation. BP in hospital ranged from 137/69 to 169/77 mmHg. She was discharged the following day.*

*She continued to be followed in the Hypertension Clinic. A mean home BP series 1-year post-RDN showed an average BP of 171/93 mmHg. A clonidine patch was offered but the patient declined, given past intolerance of oral clonidine. At last follow-up, 21 months post-procedure, BP was 150/85 mmHg and she was tolerating nifedipine 30 mg every other day reasonably well, experiencing intermittent flushing and chest tightness, attributed to the medication.*

Both cases illustrate the types and complexity of cases that are being sent for RDN here in Canada. While Case One exhibited a dramatic response following RDN, much greater than the mean reduction of 4.4/2.6 mmHg found in sham-controlled RCTs,^20^ the second case demonstrated lack of BP reduction. While these cases help frame RDN in a clinical context, they are, by nature, anecdotal, and generalized conclusions cannot be drawn from their outcomes. They nevertheless do underscore the variability in response that is seen with this procedure. This illustrates the critical need to be able to pre-identify responders.

## Implementation of RDN in clinical practice

Given the lack of hard outcome data and the, on average, modest efficacy, RDN is currently best reserved as a later-stage intervention to consider when others have failed. Not only is the BP-lowering efficacy of more conventional first-line therapies greater, but their impact on reducing cardiovascular morbidity and mortality much more clearly established.^21^

### Candidate selection

RDN should be considered through shared decision-making as a potential treatment option for interested patients willing to undergo the procedure when BP cannot be controlled by hypertension specialists through more conventional non-pharmacologic and pharmacologic options, specifically:

1. **Resistant/refractory hypertension:** Patients in whom BP remains uncontrolled after the resistant hypertension algorithm has been applied (Figure 3) and secondary causes have been ruled out or addressed. Although approximately 250,000 Canadians have treatment-resistant hypertension, it is anticipated that, after a structured approach is applied, only a small fraction would remain as candidates for RDN.
2. **MDIS with moderately or severely elevated BP (i.e., BP≥160/100 mmHg):** Patients with multiple drug intolerances and BP levels that remain uncontrolled could, in theory, be the largest candidate population for RDN treatment. However, given their general, and usually idiosyncratic, intolerance of disparate pharmacotherapies that often results in these patients remaining untreated, it remains to be seen what proportion would instead be interested in a medical procedure. General surveys of patients with hypertension (i.e., not specifically patients with MDIS) have indicated that reduction in systolic BP is the most important driver of patient preference and that 38% of untreated patients would prefer undergoing RDN to initiating pharmacotherapy.^45^^,^^46^ The minimally invasive nature and excellent safety profile of RDN may be viewed favorably by patients with MDIS. Shared decision-making is critical so that patients feel comfortable with their choice.^46^^,^^47^

Given that responders cannot currently be predetermined, it is reasonable to prioritize RDN for patients who may derive greater cardiovascular risk reduction benefit from relatively modest reductions in BP (i.e., resistant or severe hypertension, patients with pre-existing cardiovascular disease, diabetes or multiple cardiovascular risk factors, chronic kidney disease, or a 20% estimated 10-year risk of a cardiovascular event). Conversely, the procedure should be avoided in patients with advanced frailty, limited life expectancy, or anatomically related contraindications (such as renal artery stenosis or aneurysm). Notably, major trials published to-date have excluded patients with significant renal dysfunction (estimated glomerular filtration rate <40 mL/min/1.73 m^2^).^8^^,^^16^^,^^20^ Although emerging data support performing renal denervation in patients on dialysis, no center in Canada is currently performing RDN in this subgroup.^48^

### Implementation considerations

1. RDN should be performed within specialized programs only and patients should be co-evaluated by a hypertension specialist and interventionalist. The role of the hypertension specialist is to evaluate suitability for the procedure; exclude pseudohypertension and secondary causes; administer adherence counseling; and optimize non-pharmacologic and pharmacologic regimens. Careful application of these steps will likely limit use of RDN to a fraction of patients evaluated by hypertension specialists. This will ensure that the procedure is performed in suitable candidates, facilitating graded uptake and manageable procedure volumes.
2. Interventionalists are responsible for ensuring that the patient is a technically suitable candidate, performing the procedure, and follow-up of procedure-related complications. They should be trained in performing RDN and should ideally have expertise in managing potential local complications of RDN such as access site challenges and renal artery dissection, perforation, or stenosis. Discussion of potential cases with other hypertension specialists in a case conference setting is helpful to ensure there is agreement on proceeding.
3. If not recently done as part of the secondary hypertension work-up, CT angiography is performed at some CHESS centers prior to the procedure to delineate anatomy, assess procedure feasibility, and exclude contraindications such as renal artery stenosis. In this way, unnecessary invasive angiography is avoided if contraindications to renal denervation are identified on the CT angiography. It also optimizes pre-procedural planning for the RDN procedure itself.
4. Given that RDN is a relatively new procedure and results can be operator and program-dependent, centers should collect a minimal core dataset to evaluate their outcomes pre- and post-RDN. We recommend that this be a requirement of initial and ongoing funding until local effectiveness has been confidently established. This should include procedural complications, BP (24-h ambulatory BP monitoring is preferred although mean BP from a 7-day home BP series is acceptable), creatinine/estimated glomerular filtration rate, and urine albumin-creatinine ratio measured at baseline and repeated at 6-month intervals. Although adjustments to the medication regimen may cloud assessment of the impact of RDN on BP, it is not our practice to avoid making medication adjustments, if warranted. Tabulation of the number of “standard doses” of drugs (i.e., adding number of drugs used weighted by their doses expressed as a fraction of full-strength dose equivalence) is a useful means to account for changes in the drug regimen.
5. Publicly funded fee codes do not currently exist in any Canadian province as of mid-2025. CHESS centers currently performing RDN are doing so through dedicated, self-limited programmatic funding. The Medtronic Symplicity Spiral RDN system is the only option currently approved in Canada and that the per-patient cost of performing RDN using this system is estimated to be $13,500 ($6,000 device cost and $7,500 procedural cost that includes cardiac catheterization laboratory time, medical supplies, and interventionalist fees).^33^ Given that, for reasons stated above, RDN is initially ideally offered through multidisciplinary specialist programs, it seems prudent to limit public funding to designated programs within each province, and expand funding if supported by the data.
6. Some CHESS centers are admitting patients for overnight observation, although we acknowledge this is not the practice in other jurisdictions.

## Conclusions and future directions

In summary, RDN is a minimally invasive procedure that, in sham-controlled trials, safely and effectively lowers BP throughout the 24-h period, but the magnitude of effect is modest, and it is currently not possible to definitively pre-identify responders. Accordingly, we suggest that it be implemented in controlled fashion as a later-stage intervention when conventional therapies have failed. Patients with resistant/refractory hypertension or those with uncontrolled hypertension and MDIS, especially if baseline cardiovascular risk is high, are ideal candidates as they are the most likely to benefit from BP reduction. Use should be limited to designated hypertension specialist centers and a multi-disciplinary approach consisting of a hypertension specialist and interventionalist should be employed, with a hypertension specialist advancing potential candidates only after they have been properly evaluated and managed through a structured approach. Given the operator-dependent nature of the procedure, experienced interventionalists should perform all procedures and, at minimum, out-of-office BP and renal outcomes should be collected to document efficacy and durability of response. We would support a sequential program of public funding for this treatment, initially provided for a limited number of cases in a limited number of specialist centers across Canada, followed by incremental expansion if supported by positive results. Although this approach will limit the number of procedures performed, it will optimize RDN deployment to patients who are potentially most likely to benefit.

## Notes

RP has received honoraria for speaking from Medtronic and is past Chief Executive Officer of mmHg Inc., a digital health provider of software solutions, including for hypertension care (recently acquired by Myant Medical). MD has received honoraria for speaking and has served on advisory board meetings for Medtronic. NK has no disclosures. ST has received honoraria from Medtronic for education sessions, with content developed independent of the sponsor. JR has conducted clinical trials with Astra Zeneca and is also a shareholder of mmHg Inc. and Myant Medical. MM is a consultant for Medtronic and is supported by the Heart & Stroke Foundation Polo Chair in Cardiology at the University of Toronto.

This manuscript was sent to Guest Editor, Charles T. Stier Jr, PhD for editorial handling and final disposition.
